# Comprehensive analysis of gene expression and DNA methylation data identifies potential biomarkers and functional epigenetic modules for lung adenocarcinoma

**DOI:** 10.1590/1678-4685-GMB-2019-0164

**Published:** 2020-06-01

**Authors:** XiaoCong Wang, YanMei Li, HuiHua Hu, FangZheng Zhou, Jie Chen, DongSheng Zhang

**Affiliations:** 1 Hubei University of Medicine, Department of Oncology, Suizhou Hospital, Suizhou, Hubei, China; 2 Hubei University of Medicine, Department of ICU, Suizhou Hospital, Suizhou, Hubei, China

**Keywords:** DNA methylation, biomarkers, lung adenocarcinoma, cancer diagnosis, prognosis

## Abstract

Lung cancer has one of the highest mortality rates of malignant neoplasms. Lung adenocarcinoma (LUAD) is one of the most common types of lung cancer. DNA methylation is more stable than gene expression and could be used as a biomarker for early tumor diagnosis. This study is aimed to screen potential DNA methylation signatures to facilitate the diagnosis and prognosis of LUAD and integrate gene expression and DNA methylation data of LUAD to identify functional epigenetic modules. We systematically integrated gene expression and DNA methylation data from The Cancer Genome Atlas (TCGA) and Gene Expression Omnibus (GEO), bioinformatic models and algorithms were implemented to identify signatures and functional modules for LUAD. Three promising diagnostic and five potential prognostic signatures for LUAD were screened by rigorous filtration, and our tumor-normal classifier and prognostic model were validated in two separate data sets. Additionally, we identified functional epigenetic modules in the TCGA LUAD dataset and GEO independent validation data set. Interestingly, the MUC1 module was identified in both datasets. The potential biomarkers for the diagnosis and prognosis of LUAD are expected to be further verified in clinical practice to aid in the diagnosis and treatment of LUAD.

## Introduction

Lung cancer has one of the highest incidence and mortality rates of neoplasms and can be classified into small cell lung cancer and non-small cell lung cancer (NSCLC), NSCLC consists of adenocarcinoma, squamous cell carcinoma, large cell carcinoma and other types (Travis, 2011). Among them, adenocarcinoma is the most common subtype (Liu *et al.*, 2000). Despite treatment with surgery followed by radiotherapy or chemotherapy, many patients still have poor clinical outcomes (Perez *et al.*, 2014; Ramnath *et al.*, 2013; Verdecchia *et al.*, 2007). Hence, early diagnosis and treatment are the key to reducing the mortality of lung cancer. To date, no widely used DNA methylation markers have been identified for the early diagnosis and prognosis of lung adenocarcinoma (LUAD).

According to a previous study, alterations in DNA methylation (DNAm) appear to mark preneoplastic normal cells that later transform and become enriched in tumors (Teschendorff *et al.*, 2016), which indicates that DNAm could act as biomarkers for the diagnosis of early cancer. Studies on DNAm in lung cancer strongly suggest that the analyses of DNA methylation profiles will be of great utility both for understanding the molecular basis of lung cancer development (Toyooka *et al.*, 2001, 2003, 2004; Virmani *et al.*, 2002), and for developing epigenetic signatures for lung cancer (Shi *et al.*, 2017; Walter *et al.*, 2018). Recently, some studies have developed new biomarker screening algorithms for cancer diagnosis and prognosis (Wei *et al.*, 2015; Hao *et al.*, 2017), and algorithms to integrate DNA methylation and gene expression data to better understand tumor biology (Jiao *et al.*, 2014).

In this study, the gene expression and DNA methylation data of LUAD patients were systematically integrated and analyzed. We screened three potential DNA methylation signatures for early diagnosis and five prognostic gene expression signatures for LUAD. The tumor-normal classification model and prognostic model were validated in two separate data-sets. In addition, we also identified functional epigenetic modules (FEMs) in The Cancer Genome Atlas (TCGA) LUAD data set and Gene Expression Omnibus (GEO) independent validation data set. The MUC1 module was identified in both data-sets. The potential biomarkers identified in this study are expected to be further validated and may aid decision for diagnosis and treatment of LUAD.

## Material and Methods

### Dataset

#### Gene expression data analysis

We downloaded the RNA-seqV2 sequencing data (level 3, normalized count) and the corresponding clinical data of LUAD patients from the UCSC Xena database (http://xena.ucsc.edu), including 553 gene expression samples (tumor: 495, normal: 58). The screening criteria for significantly differentially expressed genes were as follows: 1.5-fold change and corrected P-value < 0.05 (independent *t*-test and p-value was adjusted by Benjamini/Hochberg correction method.

### DNA methylation data analysis

We downloaded the methylation data (level 3, Methylation 450K) and the corresponding clinical data of LUAD patients from Xena (http://xena.ucsc.edu), including 492 samples (tumor: 460, normal 32) for methylation data analysis. The methylation level of each gene was calculated by defining the average methylation level of probes within gene promoter area (TSS1500, 1stExon, TSS200, 5’ UTR), as the methylation level of the gene (TSS 1500 and TSS 200 represents 1500 bp and 200 bp downstream from the transcription start site, 1stExon represents the first exon). The screening criteria for significantly differentially methylated genes were as follows: delta Beta > 0.2 and corrected P-value < 0.05(independent *t*-test plus Benjamini/Hochberg method).

### Validation dataset

DNA methylation 450K chip data (series_matrix.txt) and gene expression data were downloaded from the NCBI-GEO database (http://www.ncbi.nlm.Nih.Gov/geo/), including GSE39279 (Sandoval *et al.*, 2013), GSE52401 (Shi *et al.*, 2014), GSE66836 (Bjaanaes *et al.*, 2016), GSE75037 (Girard *et al.*, 2016), GSE56044 (Karlsson *et al.*, 2014), GSE50081 (Der *et al.*, 2014) and GSE42127 (Tang *et al.*, 2013).

### Construction of diagnostic classifier

First, the recursive features elimination method was used to screen diagnostic probes from those 24,116 differentially methylated CpG sites. Then, probes were used to build the logistic regression function in the Python Sklearn package (version 0.19, http://scikit-learn.org/stable/index.html). The parameters were all default parameters, and the model was trained with the TCGA data. Cox proportional hazards model was built based on the screened genes and survival analysis was performed for all patients (Python lifeline 0.11.1 (http://lifelines.readthedocs.io/en/latest/index.html)), and validated in a separate data-set.

### Construction of prognostic model

We first preprocessed the GSE50081 and GSE42127 datasets downloaded from GEO. The expression of each gene was the average expression level of the corresponding probes. Then genes from the 469 differentially expressed genes were selected with cox regression analysis and log-rank test (the criterion was as follows: false discovery rate (FDR) of cox regression ≤ 0.05 and FDR of log-rank test ≤ 0.05). Then, the cox proportional hazards model was constructed with five selected genes, and survival analysis was performed for all patients.

### Gene enrichment analysis

Gene Ontology (GO) and Kyoto Encyclopedia of Genes and Genomes (KEGG) pathway enrichment analyses were performed using a web-based gene annotation tool, DAVID (Huang *et al.*, 2009a b).

### FEMs analysis

The FEM algorithm (Jiao *et al.*, 2014) is a functional supervised algorithm that uses a network of relations between genes (in our case a protein-protein interaction (PPI) network) to identify subnetworks where a significant number of genes are differentially methylated and differentially expressed. The association is measured at both the DNA methylation and gene expression levels. The algorithm thus consists of two main parts: (i) construction of an integrated network in which the associations with the phenotype are encapsulated as weights on the network edges, and (ii) inference of the FEMs as heavy subgraphs on this weighted network.

We first constructed a PPI network by integrating the InBio (Li *et al.*, 2017) and BioPlex (Huttlin *et al.*, 2015) databases. Then, we conducted FEM with the FEM algorithm by integrating the gene expression and DNA methylated data from both the TCGA and GEO datasets.

### Ethical Approval

This article does not contain any studies with human participants or animals performed by any of the authors.

## Results

### Analysis of differentially expressed genes and differentially methylated genes

**Figure 1:**
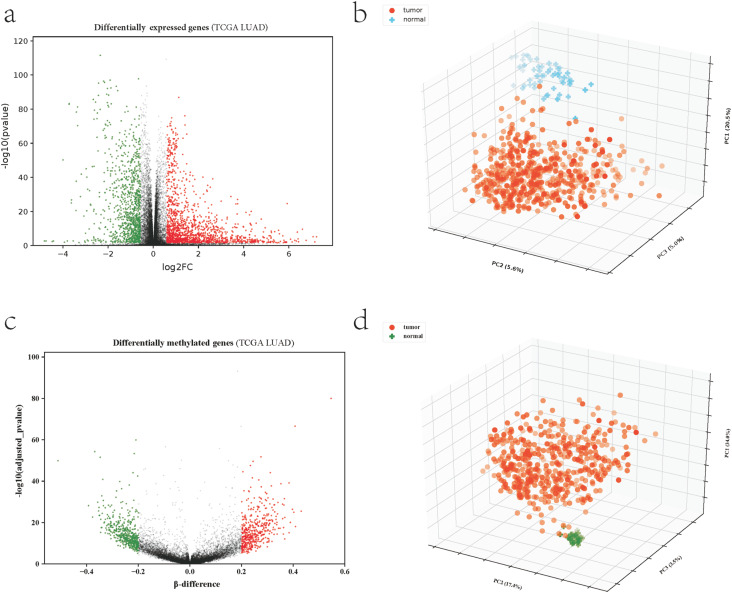
Differential expression and differential methylation analyses. (a) Volcano plot of differentially expressed genes. (b) PCA of differentially expressed genes. (c) Volcano plot of differentially methylated genes. (d) PCA of differentially methylated genes.

A total of 2469 differentially expressed genes were obtained, including 1457 upregulated and 1012 downregulated genes (Figure 1a). Principal component analysis (PCA) (Figure 1b) indicated that 1324 differentially expressed genes could effectively distinguish tumor samples from normal samples. A total of 24,116 differentially methylated CpGs retained, mapping to 981 genes, including 472 hypermethylated genes and 509 hypomethylated genes (Figure 1c). PCA indicated that the 981 differentially methylated genes could significantly separate the normal samples from the tumor samples (Figure 1d).

### Diagnostic classifier effectively distinguishes tumor samples

**Figure 2:**
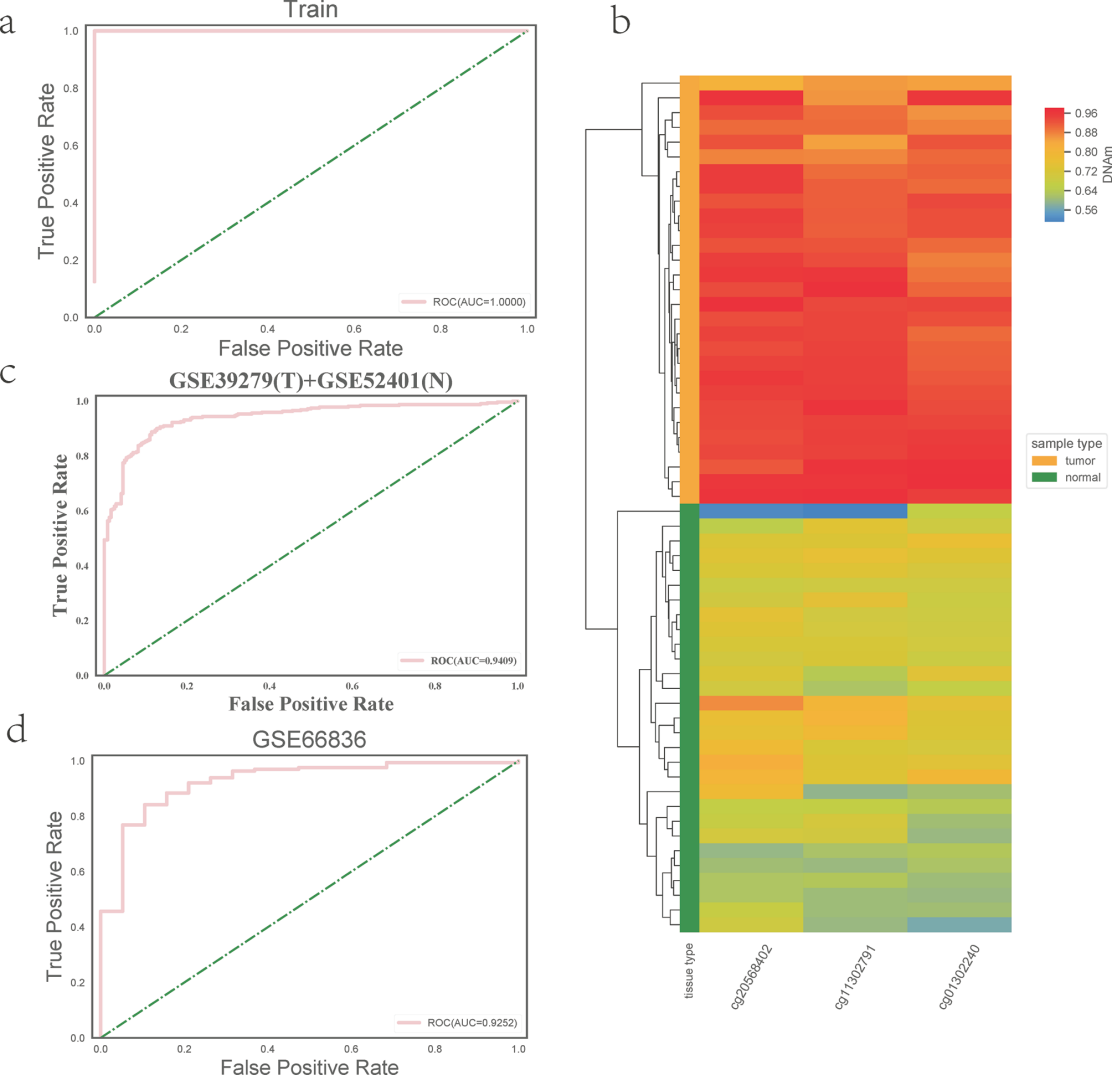
Screening of methylation markers for lung adenocarcinoma and the construction and validation of the diagnostic classifier. (a) The ROC curve of the logistic regression model. (b) Unsupervised clustering map of the methylation profile for the three DNA methylation markers. (c, d) ROC curves in the independent validation datasets.

After feature selection with recursive feature elimination (see Material and Methods), three probes (cg20568402, cg11302791 and cg01302240, see Table 1) remained. The logistic regression model constructed with these three probes performed well in the TCGA training dataset (Figure 2a, area under the curve (AUC) > 0.99). The unsupervised cluster map of the DNA methylation level of these 3 probes could clearly distinguish tumor samples from normal samples (Figure 2b), indicating that the selected three probes can be used as potential biomarkers for the diagnosis of LUAD.

**Table 1:** Detailed information of three methylation markers (probes) for LUAD diagnosis.

Probe	GeneID	Gene Symbol	Relation To Island	Group
cg20568402	55208	DCUN1D2	OpenSea	Body
cg11302791	54984	PINX1	OpenSea	Body
cg01302240	5998	RGS3	OpenSea	TSS200; Body

To further verify the repeatability of our feature selection method and classifier, we verified our model with GEO datasets (GSE39279, GSE52401 and GSE66836). As shown in Figure 2c and d, the results from the independent validation datasets are also very good (AUC > 0.92), which further indicated the reliability and accuracy of our method and model. In conclusion, our identified potential signatures may be helpful in distinguishing LUAD samples from normal samples, although further verification is needed.

### Prognostic model significantly predicts the outcome of LUAD

We first screened five genes from all of the differentially expressed genes (see Material and Methods). A cox proportional hazards model was constructed with the five selected genes (COL6A6, WFIKKN2, PLA2G1B, UMODL1 and CNGA3, see Table S1) from the TCGA LUAD dataset. Of these genes, PLA2G1B was reported to be associated with smoking-related lung adenocarcinoma (Liu *et al.*, 2016), and UMODL1 may drive lung adenocarcinoma metastasis by involving the G-protein coupled receptor protein signaling pathway (Tan *et al.*, 2016). Then, survival analysis was performed for all patients. Finally, the tumor patients were divided into high-risk and low-risk groups, which were verified in the independent data-sets GSE50081 and GSE42127. A summary of the patients in the training and validation datasets for the five-gene-based classifier is listed in Table 2.

**Table 2:** Characteristics of patients by the five-gene-based classifier assessment set.

	TCGA LUAD (N=574)	GSE50081 (Adenocarcinoma N=127)	GSE42127 (Adenocarcinoma N=133)	GSE42127 (Squamous N=43)
Age (Years, mean ± std)	65.52 ± 9.91	68.73 ± 9.71	65.76 ± 10.29	68.11 ± 7.76
Gender				
MALE	238	62	65	18
FEMALE	272	65	68	25
Stage				
I	5	0	0	0
IA	132	36	32	10
IB	134	56	57	13
II	1	0	0	0
IIA	50	7	6	3
IIB	70	28	16	7
IIIA	73	0	7	6
IIIB	11	0	13	4
IV	26	0	1	0
Survival status				
Alive	317	76	90	22
Dead	181	51	43	21
Survival time (Months, mean ± std)	30.41 ± 30.04	42.39 ± 27.66	49.67 ± 31.70	53.53 ± 34.45

N indicates the number of tumor samples.

**Figure 3:**
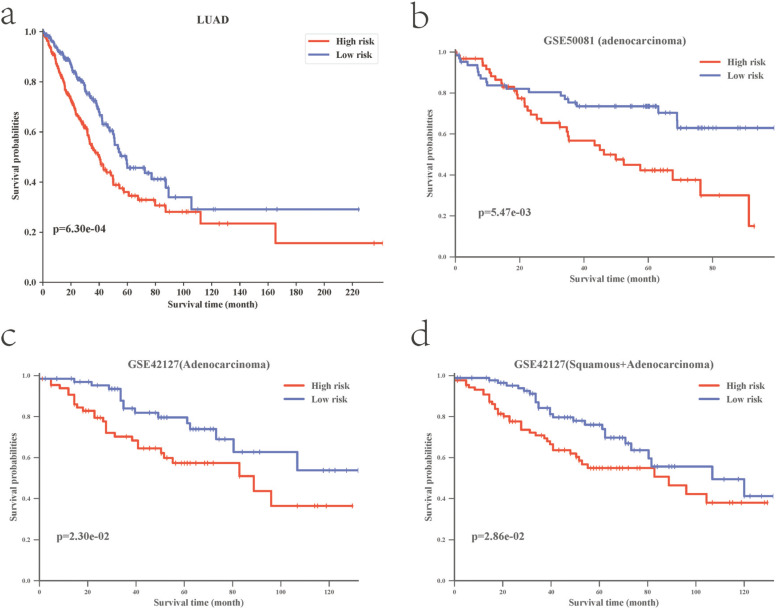
Screening of the prognostic markers for lung adenocarcinoma and the construction of the prognostic classifier. (a) K-M curve in the TCGA training dataset. (b,c,d) K-M curve in the independent validation dataset.

As shown in Figure 3, whether in the TCGA training dataset (Figure 3a) or independent validation datasets (Figure 3b–d), the five genes can significantly divide patients into high-risk and low-risk groups (P-value < 0.05), and the prognosis of patients in the high-risk group is significantly worse than that of patients in the low-risk group. In addition, the five potential prognostic markers screened from the LUAD can also significantly divide all NSCLC samples (including lung squamous carcinoma) into high- and low-risk groups (P-value < 0.01, Figure 3d), which further illustrates the repeatability of our classifier. We further performed survival analysis with regard to the five-gene-based classifier in subsets of patients with different clinical variables in the TCGA LUAD dataset. When stratified by clinical variables (sex, age, and pathologic stage), the five-gene-based classifier was still a statistically significant prognostic model (Figure S1).

### The FEM MUC1 was identified in LUAD

**Figure 4:**
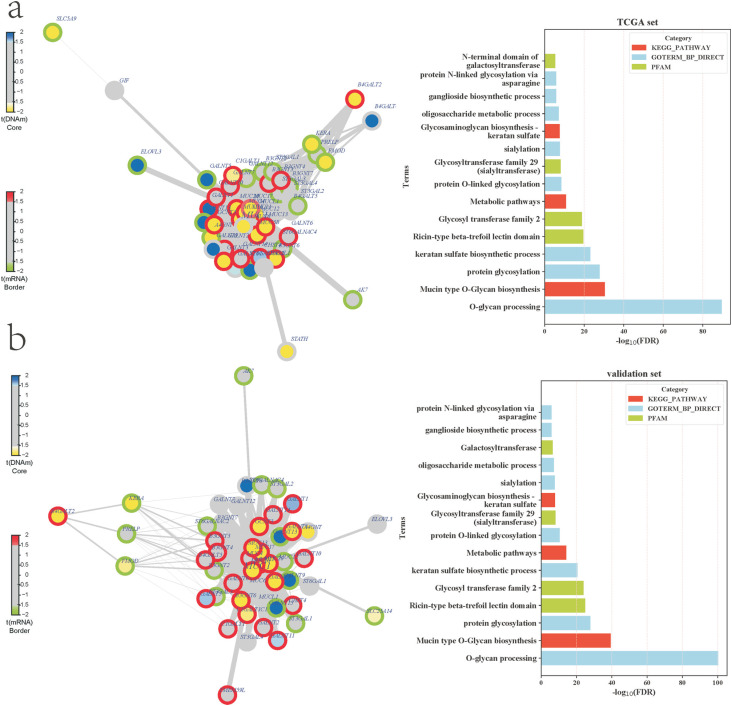
Functional epigenetic modules of lung adenocarcinoma. (a) MUC1 module identified in TCGA (left) and enrichment analysis results (right). (b) MUC1 module identified in the GEO validation set (left) and enrichment analysis (right). The node color indicates the DNA methylation difference (blue indicates high methylation, and yellow indicates low methylation), and the edge color indicates differentially expressed genes (red represents genes with high expression level in tumors and green represents genes with low expression in tumors).

We implemented the FEM algorithm (Jiao *et al.*, 2014) to integrate gene expression and DNA methylation data to perform FEM analysis, and four different functional modules were identified in the TCGA dataset: (MUC1) (Figure 4a), ADCY8, CAOLEC10 and WNT3A (Figure S2). Then, in the validation data set (GSE75037 and GSE56044), three modules were identified: MUC1 (Figure 4b), GSTMS and OTX1 (Figure S2), of which, the MUC1 module was identified in both of the datasets. The MUC1 modules identified in the two datasets were significantly overlapping (45 overlapping genes, P-value < 0.01, hypergeometric test) (Figure 4 and Table S2).

The genes in the MUC1 module were enriched in the ricin lectin domain structure and participated in biological processes such as O polysaccharide processing, protein glycosylation, keratin sulfate biosynthesis, mucin O polysaccharide biosynthesis, and sheath sugar lipid biosynthesis as well as metabolic signaling pathways (Figure 4). Pro-oncogenic mucin MUC1 was reported to contribute to smoking-induced lung cancers that are driven by inflammatory signals from macrophages (Xu *et al.*, 2014). A previous study showed that overexpression of MUC1 induces epithelial-mesenchymal transition and promotes the metastasis of lung cancer cells (Xue *et al.*, 2017). In addition, MUC1 was reported to be useful in predicting prognosis in NSCLC patients (Zhu *et al.*, 2014) and may contribute to the treatment of patients with NSCLC resistant to EGFR kinase inhibitors (Kharbanda *et al.*, 2014), indicating that our identified functional epigenetic module MUC1 may play an important role in the development and prognosis of LUAD.

## Discussion

LUAD is one of the most common neoplasms, and the early diagnosis of LUAD has always been challenging. DNA methylation changes have been reported to occur early in carcinogenesis (Teschendorff *et al.*, 2016), and DNA methylation analysis seems to be a promising strategy in cancer diagnosis. This study used the data of the TCGA LUAD and GEO public datasets, performed an integrative analysis of gene expression and DNA methylation data, and selected three potential DNA methylation biomarkers for the diagnosis of LUAD. All three CpGs are differentially variable and differentially methylated CpGs (DVMCs) in the TCGA LUAD dataset (Figure S3). DVMC was defined by Teschendorff *et al.* (2016) and could be useful to identify field defects. In addition, five potential prognostic gene expression signatures selected from the differentially expressed genes could be used to predict the outcome of LUAD patients. We also performed stratification analysis, in which, different clinical variables (sex, age and stage) were evaluated separately and our prognostic model could also divide tumor patients into high-risk and low-risk groups with significantly different outcomes. Finally, we identified the functional epigenetic module MUC1, which plays a certain role in LUAD, in both the TCGA and GEO datasets. The potential DNA methylation biomarkers identified in this study may be used to design appropriate methylation targeted therapies.

## Supplementary Material

The following online material is available for this article:

Figure S1 Kaplan Meier curves of the five-genes-based classifier between high-risk and low-risk groups stratified by clinical variables (sex, age, and pathologic stage).

Figure S2 Other functional epigenetic modules identified in the TCGA dataset (ADCY8, CAOLEC10 and WNT3A) and the validation data set (GSTMS and OTX1).

Figure S3 Illustration of differentially variable and differentially methylated CpGs (DVMCs) for the three diagnostic markers in the TCGA LUAD dataset.

Table S1 Table of cox proportional hazards model result for the selected five genes used to construct the prognostic model.

Table S2 Information for the members of MUC1 modules identified in the TCGA and independent validation datasets.
