# Supplementary material for: Comprehensive analysis of gene expression and DNA methylation data identifies potential biomarkers and functional epigenetic modules for lung adenocarcinoma
Source: Genet Mol Biol. 2020 Jun 1;43(3):e20190164. doi: 10.1590/1678-4685-GMB-2019-0164 (PMC7299274; doi:10.1590/1678-4685-GMB-2019-0164)
Supplement: Supplementary file 1 [file 1415-4757-GMB-43-3-e20190164-suppl04.pdf]

Supplementary Material to “Comprehensive analysis of gene expression and DNA methylation data identifies potential biomarkers and functional epigenetic modules for lung adenocarcinoma”

Table S1 - Cox proportional hazards model result for the five selected genes.

GeneSymbol	Pvalue (univariate cox)	FDR (univariate cox)	Pvalue (logRank)	FDR (logRank)	DE_type
**COL6A6**	0.001951968	0.026359258	1.233E-05	0.0137002	down-regulated
**WFIKKN2**	0.001032216	0.01831425	2.079E-07	0.0020786	down-regulated
**PLA2G1B**	0.003005784	0.033471986	8.924E-06	0.0127479	down-regulated
**UMODL1**	0.003547928	0.036351725	1.856E-05	0.0185628	up-regulated
**CNGA3**	0.000737133	0.014831646	5.939E-06	0.0098988	up-regulated
