# Supplementary material for: Comprehensive analysis of gene expression and DNA methylation data identifies potential biomarkers and functional epigenetic modules for lung adenocarcinoma
Source: Genet Mol Biol. 2020 Jun 1;43(3):e20190164. doi: 10.1590/1678-4685-GMB-2019-0164 (PMC7299274; doi:10.1590/1678-4685-GMB-2019-0164)
Supplement: Supplementary file 3 [file 1415-4757-GMB-43-3-e20190164-suppl02.pdf]

Supplementary Material to "Comprehensive analysis of gene expression and DNA methylation data identifies potential biomarkers and functional epigenetic modules for lung adenocarcinoma"

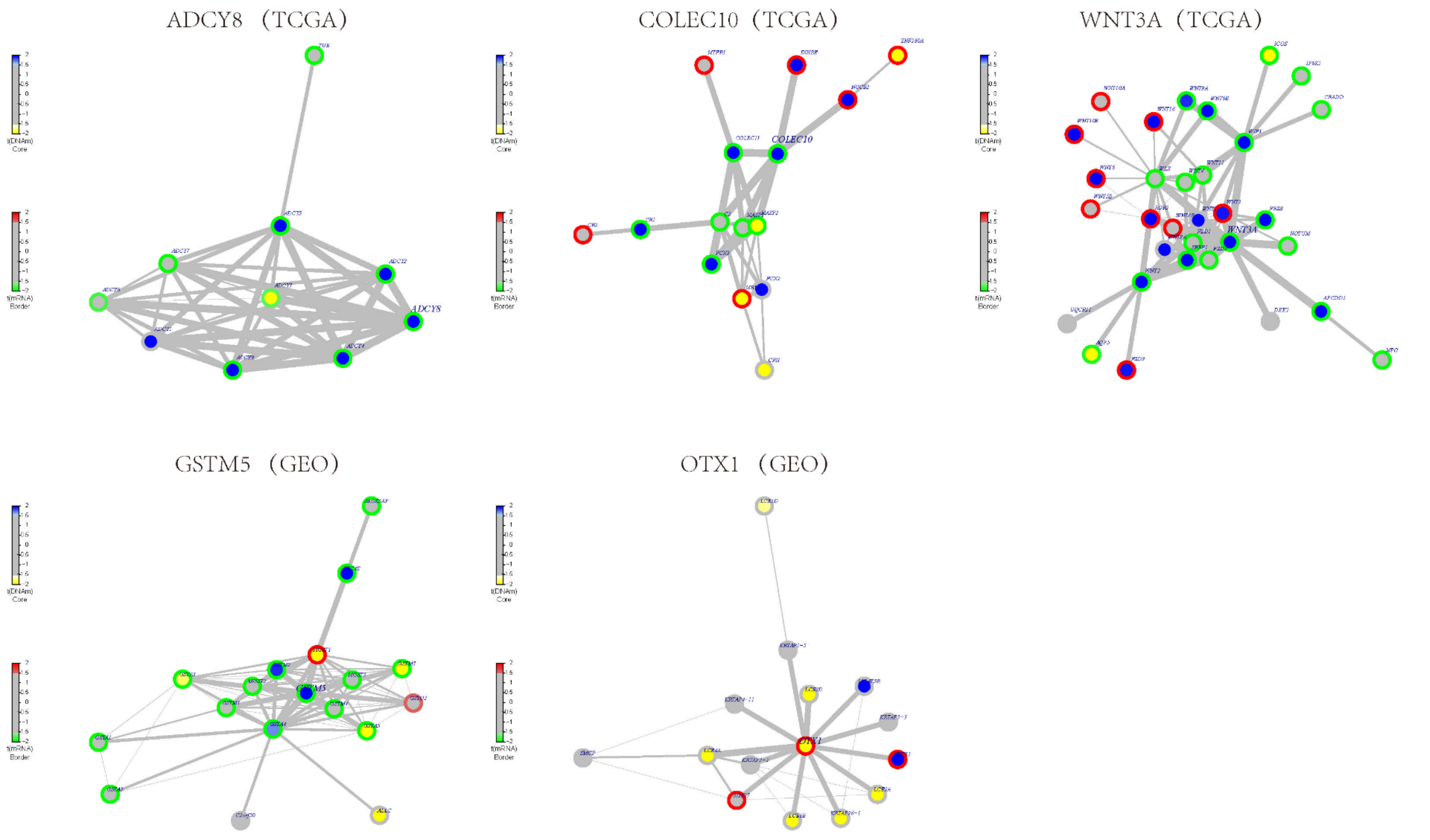

Figure S2 - Other functional epigenetic modules identified in the TCGA dataset (ADCY8, CAOLEC10 and WNT3A) and the validation data set (GSTMS and OTX1).
