# Supplementary material for: Comprehensive analysis of gene expression and DNA methylation data identifies potential biomarkers and functional epigenetic modules for lung adenocarcinoma
Source: Genet Mol Biol. 2020 Jun 1;43(3):e20190164. doi: 10.1590/1678-4685-GMB-2019-0164 (PMC7299274; doi:10.1590/1678-4685-GMB-2019-0164)
Supplement: Supplementary file 4 [file 1415-4757-GMB-43-3-e20190164-suppl05.pdf]

Supplementary Material to "Comprehensive analysis of gene expression and DNA methylation data identifies potential biomarkers and functional epigenetic modules for lung adenocarcinoma"

Table S2 - Information for the members of MUC1 modules identified in the TCGA and independent validation datasets.

TCGA MUC1 Module (50 genes)						
**EntrezID**	**Symbol**	**Stat (DNAm)**	**P (DNAm)**	**Stat (mRNA)**	**P (mRNA)**	**Stat (Int)**
4582	MUC1	-19.72435052	3.29E-64	0.481086225	0.630638192	19.9768777
79695	GALNT12	0.854467549	0.393262704	-1.823792189	0.068702324	1.811795077
51146	A4GNT	-5.873088815	7.89E-09	-5.563696879	4.05E-08	0
10678	B3GNT2	1.149779707	0.250794355	-12.12455874	2.74E-30	7.514086954
6487	ST3GAL3	-0.244171964	0.807199639	-3.959479295	8.45E-05	0
93010	B3GNT7	0.029446762	0.976520243	-2.863106858	0.004348008	1.532321392
51809	GALNT7	0.256338498	0.797796843	14.19401362	2.02E-39	0
63917	GALNT11	1.525274614	0.127834484	0.36626023	0.714305597	0
10610	ST6GALNAC2	2.014369892	0.044515514	-10.49612518	1.06E-23	7.523895394
2589	GALNT1	5.848355086	9.07E-09	4.633776083	4.45E-06	0
27090	ST6GALNAC4	0.5580073	0.577093744	-2.940977079	0.003403424	2.10175682
6484	ST3GAL4	-0.612237796	0.540663863	0.635219983	0.525537596	0.945671385
10331	B3GNT3	-1.148831036	0.251185021	19.09724582	2.47E-63	11.17317441
55568	GALNT10	-1.103159784	0.270498185	1.992712433	0.046765091	2.149155308
6482	ST3GAL1	-0.107392461	0.914521504	-2.802630065	0.005239657	0
114805	GALNT13	5.928055897	5.78E-09	-10.88122427	3.34E-25	11.63972392
192134	B3GNT6	-8.238346013	1.60E-15	10.6230377	3.43E-24	13.81448922
2590	GALNT2	0.987850658	0.323712355	8.704814031	3.36E-17	0
79369	B3GNT4	1.036638984	0.300414412	9.756971973	6.49E-21	0
9245	GCNT3	-5.77759239	1.35E-08	12.6092636	2.34E-32	12.39632627
9334	B4GALT5	0.375668397	0.707325685	1.020970043	0.307697904	0
10164	CHST4	1.615617567	0.106819383	2.593445302	0.009743898	0
11226	GALNT6	-0.587771101	0.556956172	6.57575166	1.09E-10	4.039451681
50614	GALNT9	7.094782207	4.55E-12	-0.788528474	0.430712556	7.508689022
79623	GALNT14	0.066802602	0.946766045	11.11859453	3.79E-26	0
56913	C1GALT1	-1.754626106	0.079947036	6.624682403	8.01E-11	5.231990944
2591	GALNT3	-3.115717348	0.001942383	2.867409024	0.004290165	4.620850229
6480	ST6GAL1	0.158103927	0.87443991	0.015341494	0.98776506	0

TCGA MUC1 Module (50 genes)						
**EntrezID**	**Symbol**	**Stat (DNAm)**	**P (DNAm)**	**Stat (mRNA)**	**P (mRNA)**	**Stat (Int)**
11227	GALNT5	4.181734867	3.43E-05	-5.529868912	4.87E-08	7.084420693
6483	ST3GAL2	1.180063891	0.238546224	-7.866047664	1.82E-14	5.309034291
56667	MUC13	-7.678149493	8.80E-14	6.770999045	3.16E-11	11.23231746
200958	MUC20	-5.007096298	7.72E-07	7.860256336	1.90E-14	9.133026771
394263	MUC21	-4.660621537	4.07E-06	4.29297864	2.07E-05	6.914050767
94025	MUC16	-3.353332905	0.000860159	5.996486908	3.56E-09	6.500951379
4585	MUC4	-2.893160402	0.003983345	4.889457355	1.31E-06	5.459687526
727897	MUC5B	-3.542673919	0.000433905	5.305073641	1.61E-07	6.327362364
140453	MUC17	-3.031842475	0.002559134	1.583274288	0.113908679	3.86291965
118430	MUCL1	-3.108536353	0.001989265	1.383367072	0.167089084	3.834680146
4588	MUC6	-0.558369206	0.576846833	1.348806687	0.177929912	1.266371893
10071	MUC12	-0.70528865	0.480965082	-3.547088879	0.000421274	0
83401	ELOVL3	2.657306771	0.008133434	-4.727337787	2.86E-06	5.138735645
122481	AK7	0.991591197	0.321885407	-7.464307704	3.12E-13	4.909684105
5549	PRELP	0.606672243	0.544348859	-9.855240543	2.82E-21	5.779790718
8704	B4GALT2	-4.49019749	8.88E-06	15.39525314	4.91E-45	12.57132631
11081	KERA	-8.030573746	7.24E-15	-2.278615944	0.023055674	0
2331	FMOD	-2.027203243	0.043181381	-3.068191315	0.002254963	0
6779	STATH	-4.485048982	9.09E-06	0.757990158	0.448767352	4.882925916
2694	GIF	0.617313947	0.537313847	1.044369374	0.296753294	0
9331	B4GALT6	3.618929114	0.000326501	-1.211902643	0.22604731	4.255069442
200010	SLC5A9	-4.075753158	5.35E-05	-15.36749462	6.65E-45	0

GEO MUC1 Modules (51 genes)						
**EntrezID**	**Symbol**	**Stat (DNAm)**	**P (DNAm)**	**Stat (mRNA)**	**P (mRNA)**	**Stat (Int)**
4582	MUC1	-11.79384115	1.73E-22	2.227648902	0.027240941	12.47995667
79695	GALNT12	0.548353545	0.584357449	-0.674947356	0.500643253	0.756237228
374907	B3GNT8	3.183287956	0.001808879	0.147143634	0.883196266	0
51146	A4GNT	-3.278932869	0.001326337	-0.414071678	0.679353312	0
10678	B3GNT2	0.218731226	0.827190709	-10.10853916	4.86E-19	3.33215974
6487	ST3GAL3	0	1	-2.235697898	0.02669769	0.68859461
93010	B3GNT7	-1.061559325	0.290336672	0.350627094	0.726310325	1.169552416
51809	GALNT7	0.416958566	0.677372904	1.235024768	0.218557277	0

GEO MUC1 Modules (51 genes)						
**EntrezID**	**Symbol**	**Stat (DNAm)**	**P (DNAm)**	**Stat (mRNA)**	**P (mRNA)**	**Stat (Int)**
63917	GALNT11	1.575511652	0.117485747	3.915548351	0.000131228	0
10610	ST6GALNAC2	-0.511034418	0.610164115	-8.365186188	2.29E-14	0
2589	GALNT1	1.650709426	0.101129215	4.163245589	5.02E-05	0
27090	ST6GALNAC4	-1.48287428	0.140445306	-4.426088784	1.73E-05	0
29071	C1GALT1C1	-1.761446593	0.080433271	5.433385206	1.93E-07	3.434928405
6484	ST3GAL4	-0.564217784	0.573544826	-1.053449139	0.29365807	0
10331	B3GNT3	-0.817448502	0.415116689	7.719564013	1.02E-12	3.195073037
55568	GALNT10	0	1	3.913617326	0.000132193	1.205393537
6482	ST3GAL1	-0.010797802	0.991400755	-6.71943235	2.75E-10	0
114805	GALNT13	3.205698054	0.001683039	-8.921141425	7.93E-16	5.953408247
192134	B3GNT6	-2.900111461	0.004358075	8.284776331	3.69E-14	5.451821303
2590	GALNT2	-0.298559527	0.765736542	5.433979686	1.92E-07	1.972224438
79369	B3GNT4	0.080662863	0.935829876	7.561887806	2.52E-12	0
9245	GCNT3	-1.90738612	0.058600877	18.49334876	2.97E-42	7.603334708
9334	B4GALT5	-0.663153251	0.508366445	3.518097618	0.000560127	1.746726778
25789	TMEM59L	1.048600872	0.296240935	6.762454071	2.18E-10	0
26290	GALNT8	-1.90509393	0.058900796	1.867828743	0.063539286	2.480384897
10164	CHST4	-0.324784371	0.745848478	3.05418288	0.002627485	1.26547223
11226	GALNT6	-0.951439913	0.343086348	12.79216815	1.50E-26	4.891425747
50614	GALNT9	3.939328549	0.000130597	-3.942760458	0.000118323	5.153698167
79623	GALNT14	-0.953893223	0.341847519	6.355635736	1.91E-09	2.911428057
117248	GALNT15	3.308133563	0.001204871	-3.047936883	0.002679732	4.246897657
56913	C1GALT1	0.592235475	0.554686401	8.282125288	3.75E-14	0
2591	GALNT3	-10.81458672	5.26E-20	10.00417868	9.40E-19	13.89587222
6480	ST6GAL1	-0.389899785	0.697226891	-1.061724256	0.289894963	0
11227	GALNT5	1.632145357	0.10498609	4.667063747	6.24E-06	0
6483	ST3GAL2	-0.665592639	0.506810432	-1.763831362	0.079590226	0
94025	MUC16	-3.388664381	0.000921568	7.766590363	7.76E-13	5.780773024
200958	MUC20	-2.263176332	0.025225425	12.36798328	2.36E-25	6.072513288
394263	MUC21	-2.031440382	0.044178563	8.142548145	8.59E-14	4.539343965
56667	MUC13	-3.50305053	0.000624793	6.176107591	4.84E-09	5.405290723
140453	MUC17	-2.226359812	0.027652438	2.769644884	0.006247474	3.079410013
4585	MUC4	-0.730777479	0.466185151	7.63119235	1.70E-12	3.081183555
4588	MUC6	-0.89995477	0.369751504	0.429648002	0.668005943	1.032286289

GEO MUC1 Modules (51 genes)						
**EntrezID**	**Symbol**	**Stat (DNAm)**	**P (DNAm)**	**Stat (mRNA)**	**P (mRNA)**	**Stat (Int)**
10071	MUC12	-1.185970599	0.237723099	-2.057530971	0.041190816	0
118430	MUCL1	-1.141067373	0.255867855	-0.091755237	0.927002559	0
83401	ELOVL3	1.110254574	0.26886871	0.110613429	0.912055813	0
122481	AK7	-0.755593004	0.4512134	-2.439418042	0.01575801	0
9016	SLC25A14	-1.676939773	0.09587503	-2.010081722	0.046033392	0
11081	KERA	-4.043899478	8.81E-05	-2.580438662	0.010728259	0
8704	B4GALT2	-2.248553665	0.026166116	3.603516954	0.000414045	3.358436335
5549	PRELP	-1.069931197	0.286565054	-18.97072036	1.65E-43	0
2331	FMOD	-1.720909818	0.087564825	-6.2261847	3.74E-09	0
