# Supplementary material for: Comprehensive analysis of gene expression and DNA methylation data identifies potential biomarkers and functional epigenetic modules for lung adenocarcinoma
Source: Genet Mol Biol. 2020 Jun 1;43(3):e20190164. doi: 10.1590/1678-4685-GMB-2019-0164 (PMC7299274; doi:10.1590/1678-4685-GMB-2019-0164)
Supplement: Supplementary file 5 [file 1415-4757-GMB-43-3-e20190164-suppl03.pdf]

Supplementary Material to "Comprehensive analysis of gene expression and DNA methylation data identifies potential biomarkers and functional epigenetic modules for lung adenocarcinoma"

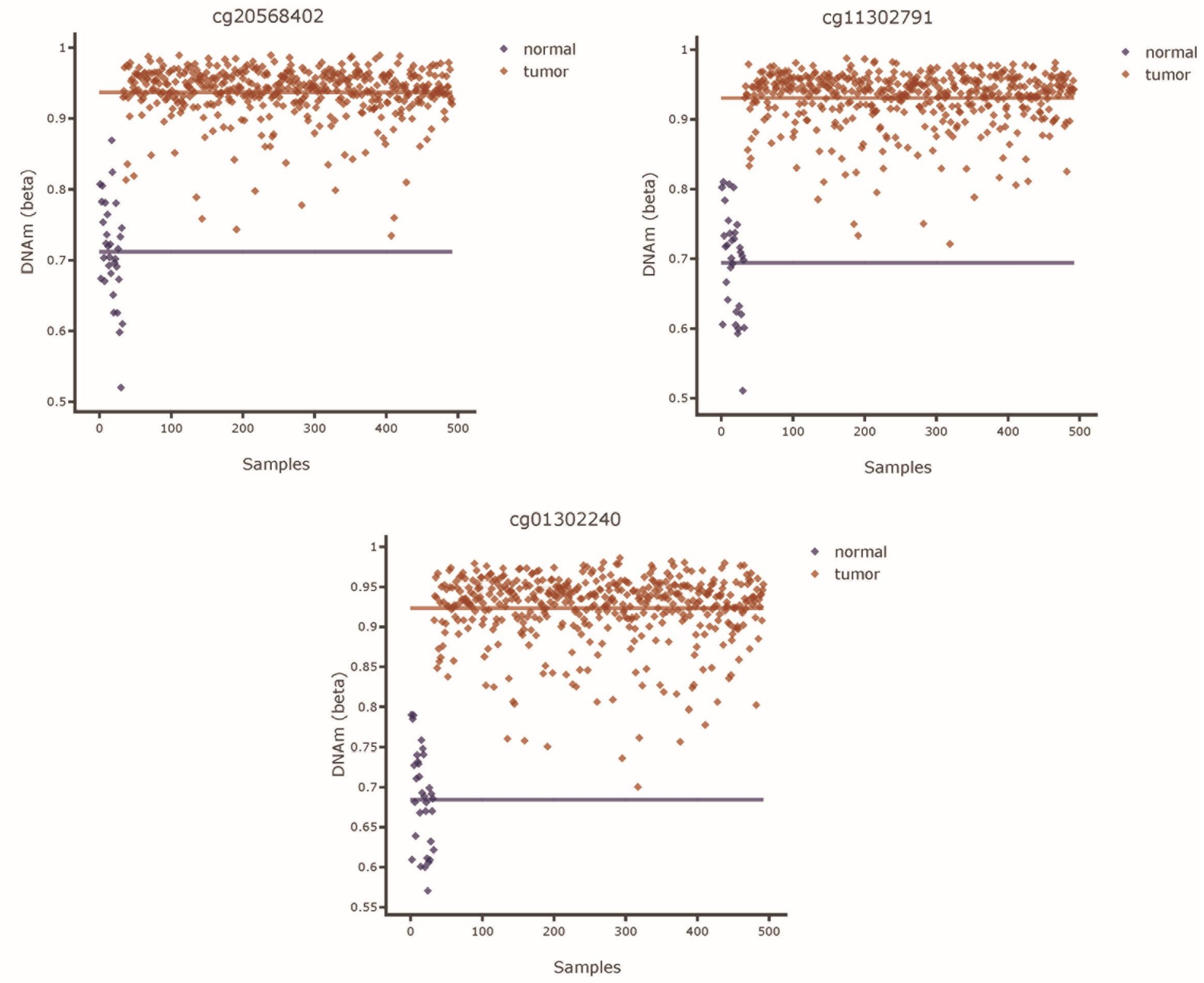

Figure S3 - Illustration of differentially variable and differentially methylated CpGs (DVMCs) for the three diagnostic markers in the TCGA LUAD dataset.
